# Waterborne Polyurethane Reinforced with SiO_2_-Modified TiO_2_: Enhanced Mechanical Properties and Retained Hydrostatic Pressure Resistance

**DOI:** 10.3390/polym18121492

**Published:** 2026-06-13

**Authors:** Shuyi Wang, Weiping Yao, Xia Lin, Yamin Xu, Kemei Pei, Yuhai Lu

**Affiliations:** 1 College of Chemistry and Chemical Engineering, Zhejiang Sci-Tech University, Hangzhou 310018, China; 2024211001047@mails.zstu.edu.cn (S.W.); yaminxu0815@163.com (Y.X.); peikemei@zstu.edu.cn (K.P.); 2 Gensen Auto Parts Inc., Ningbo 315300, China; yaoweiping@gensenauto.com (W.Y.); xialin@gensenauto.com (X.L.); 3 Hangzhou Zhuodao New Materials Co., Ltd., Hangzhou 311100, China

**Keywords:** SiO_2_/TiO_2_, waterborne polyurethane, hydrostatic pressure resistance, mechanical properties

## Abstract

Driven by the growing demand for functional textiles featuring excellent waterproofness, moisture permeability and mechanical robustness in outdoor sportswear, medical protection and technical apparel, traditional pongee—despite its desirable softness, high wrinkle resistance and good stability as an ideal substrate fabric—is severely restricted in further application by its intrinsically poor hydrostatic pressure resistance in extremely wet environments. Accordingly, we developed a modified waterborne polyurethane (WPU) coating for pongee substrates to fabricate functional textiles that maintain high hydrostatic pressure resistance while possessing good mechanical properties and increased UV absorption. In this study, by using the sol–gel method, an amorphous silicon dioxide (SiO_2_) coating layer was constructed on the surface of titanium dioxide (TiO_2_) particles, forming silica-modified titania particles (SiO_2_/TiO_2_). These SiO_2_-modified particles were subsequently physically blended with an anionic waterborne polyurethane system that had been previously modified with a polyester-type modifier A to enhance its hydrostatic pressure resistance. The resulting composite coating was designed to combine the high hydrostatic pressure resistance inherited from the modified WPU matrix, the mechanical reinforcement and increased UV absorption contributed by SiO_2_/TiO_2_, and satisfactory water repellency on fabric substrates. The results indicate that the incorporation of an appropriate amount of modifier A into the prepolymer system significantly enhances hydrostatic pressure resistance while maintaining high elongation at break. At a SiO_2_/TiO_2_ loading of 0.2 wt%, the composite film exhibits optimal comprehensive performance, characterized by superior mechanical properties, low water absorption, and static water contact angles exceeding 100° for coated fabrics. SiO_2_/TiO_2_ composite WPU coatings substantially improve hydrostatic pressure resistance across various fabrics, with 380T polyester taffeta demonstrating the best performance. This resistance remains remarkably stable after standard washing, indicating excellent wash fastness and practical applicability.

## 1. Introduction

Waterborne polyurethane (WPU) is an environmentally friendly polymer material. Through rational molecular structure design, hydrophobic functional groups can be covalently grafted into the molecular chains of waterborne polyurethane, leading to a remarkable improvement in its water resistance and hydrophobic properties [1,2,3,4,5]. The incorporation of hydrostatic pressure-resistant functional components represents a particularly noteworthy strategy in this regard [6]. Among various functional components, polyester-type polyols have been demonstrated as effective soft segments for enhancing the hydrostatic pressure resistance of waterborne polyurethanes. De Smet et al. [7] synthesized a fully bio-based high-performance waterborne polyurethane (WPU) coating utilizing polyester polyol (Priplast 3190) as the soft segment, in conjunction with polyisocyanate crosslinker (Edolan XCI), via a three-layer coating deposition process followed by high-temperature curing. The as-fabricated coating exhibited exceptional hydrostatic pressure resistance, which was well retained after hydrolysis treatment and repeated standard washing cycles. Lacruz et al. [8] selected a highly hydrophobic bio-based polyester polyol, Priplast 3238, optimized the soft-to-hard segment ratio to achieve a high elongation at break, and covalently incorporated trans-cyclohexanediol isobutyl POSS (POSS-OH) without sacrificing hydrostatic pressure resistance, thereby enabling all coated fabrics to exhibit a high level of water column values and achieve hydrostatic pressure performance comparable to that of petroleum-based coatings. Due to the outstanding properties of high-hydrostatic-pressure-resistant waterborne polyurethane, this material has found extensive applications across multiple fields, including outdoor protective clothing, tents, military equipment, and medical protective gear [9,10,11,12].

High-hydrostatic-pressure-resistant waterborne polyurethanes have garnered sustained attention from both research and industrial sectors due to their exceptional functional properties and vast market potential. Research and development in this field remains relatively limited, primarily focusing on two aspects: the selection and incorporation of high-hydrostatic-pressure-resistant functional components, and the optimization of film-forming processes. The selection and incorporation of high hydrostatic pressure-resistant components primarily involves introducing various high-performance elements—such as carbon nanotubes, nanoclay, fluorinated diols, or diamines—into the polymeric polyurethane chain segments through different methods. Jiang et al. [13] synthesized a long-chain alkyl-containing WPU-acrylate hybrid emulsion, forming a low-surface-energy hydrophobic coating on cotton fabric surfaces. This doubled hydrostatic pressure resistance, achieving fluorine-free, eco-friendly, and durable waterproofing effects. Bramhecha et al. [14] built upon the interaction between citric acid-functionalized WPU and the hydroxyl groups of cotton fabric, uniformly dispersing ultra-low doses of graphene within the WPU paste. Using a doctor blade coating method with a support roller, they formed a defect-free, non-porous, continuous composite film on the surface of the cotton fabric, achieving excellent waterproof barrier performance and good vapor management capability. Chen et al. [15] incorporated the poly(propylene oxide)-*b*-poly(ethylene oxide)-*b*-poly(propylene oxide) triblock diols (PO-EO-PO diols) into WPU and adjusted their ratio to significantly enhance hydrostatic pressure resistance while maintaining good moisture permeability. Different application methods directly determine the film’s thickness, density, and crosslinking degree, which significantly influence its hydrostatic pressure resistance. Moiz et al. [16] combined WPU with polydimethylsiloxane-trimethylated silica (PDMS-TMS) using a Pad-Knife-Pad three-layer composite coating process to build a dense, hydrophobic protective film on cotton fabric surfaces, substantially enhancing hydrostatic pressure resistance and achieving outstanding waterproofing. Patti et al. [17] innovatively introduced fluorinated polyurethane-perfluoropolyether (PU-PFPE) onto a polypropylene (PP) fabric via spraying, rather than the traditional coating or lamination method. Combined with high-temperature, high-pressure drying, this process caused the polyurethane (PU) to melt, flow, and compact the fabric pores, substantially boosting hydrostatic pressure resistance. This achieved simultaneous improvements in mechanical strength, abrasion resistance, and waterproofing without significant weight gain or alteration to the fabric’s appearance. Beyond merely enhancing hydrostatic pressure resistance, researchers also focus on improving other properties of polyurethane materials while maintaining high hydrostatic pressure resistance. These include ultraviolet (UV) aging resistance [18], biodegradability [19], corrosion resistance [20], and thermal stability [21]. Wang et al. [22] produced hydrophilic monomer itaconic acid polyethylene glycol monomethyl ether ester (IM) via esterification of itaconic acid with polyethylene glycol monomethyl ether, then introduced it via UV grafting to create waterproof and moisture-permeable WPU. This ensures the film maintains optimal flexibility while balancing high waterproofing with high moisture permeability. Feng et al. [23] incorporated the environmentally friendly phosphorus-based flame retardant ExolitOP550, a polyester diol, into the WPU backbone, achieving both flame retardancy and moderate water resistance. Zhou et al. [24] introduced polycarbodiimide (PCD) and long-chain alkyl polymer (LAP), combined with electrospinning and post-heat treatment, forming a highly hydrophobic surface, successfully producing an eco-friendly fluorine-free WPU nanofiber membrane. This film exhibits significantly enhanced hydrostatic pressure resistance while maintaining good water vapor permeability and excellent resilience. Heng et al. [25] achieved an optimal balance between key properties like hydrostatic pressure resistance and moisture permeability by blending graphene oxide (GO) with carbon nanotubes (CNTs) into WPU, imparting multifunctional properties such as heat storage, antistatic, and antibacterial characteristics to the modified membrane. Lacruz et al. [26] physically blended single-walled carbon nanotubes (SWCNT) into a polyester-based WPU matrix, obtaining multifunctional coatings with excellent electrical conductivity while retaining the original hydrostatic pressure resistance. Regarding the development of hydrostatic pressure-resistant waterborne polyurethanes, synergistic optimization of hydrostatic pressure resistance with other material properties is crucial. This approach aims to balance hydrostatic pressure resistance with fundamental properties such as mechanical strength, weather resistance, and chemical corrosion resistance. Ensuring that the material’s physical and mechanical properties, weather resistance, and solvent resistance meet practical application requirements while guaranteeing waterproofing performance represents one of the key scientific challenges for future research.

Titanium dioxide (TiO_2_) can enhance the mechanical properties [27], impart UV absorption capabilities [28], and provide antibacterial activity [29,30] of WPU. Furthermore, TiO_2_ shows satisfactory biocompatibility and is regarded as safe for routine contact in textile practical applications, making it highly suitable for textile applications. Furthermore, by modifying silicon dioxide (SiO_2_) onto the surface of TiO_2_ nanoparticles forming silica-modified titania particles (SiO_2_/TiO_2_) and physically blending them with a waterborne polyurethane system, the material’s weather resistance and photochemical stability can be synergistically enhanced. In this study, SiO_2_/TiO_2_ nanoparticles were employed as functional reinforcement and were synthesized via the sol–gel method. A waterborne polyurethane modified with polyester-type modifier A served as the matrix for physical blending and compounding, resulting in a composite coating that achieves high hydrostatic pressure resistance while maintaining enhanced mechanical strength and water resistance. Based on this, the effects of SiO_2_/TiO_2_ dosage on the stability of the blended WPU emulsion, the mechanical properties of the film, water absorption rate, and hydrostatic pressure resistance were systematically investigated. Furthermore, its application performance on different fabrics was explored, achieving synergistic optimization between waterproof breathability and mechanical properties of the WPU coating.

## 2. Materials and Methods

### 2.1. Materials

Polytetramethylene ether glycol (PTMG, Mn = 1000 g mol^−1^), polycarbonate diol (PCDL, Mn = 1000 g mol^−1^), toluene diisocyanate (TDI-80), trimethylolpropane (TMP), triethylamine (TEA), and sodium hexametaphosphate were purchased from Shanghai Macklin Biochemical Technology Co., Ltd., Shanghai, China. 2,2-Bis(hydroxymethyl)butyric acid (DMBA) was obtained from Saen Chemical Technology (Shanghai) Co., Ltd., Shanghai, China. Ethylenediamine (EDA) was sourced from Shanghai Lingfeng Chemical Reagent Co., Ltd., Shanghai, China. Acetone was supplied by Huzhou Shuanglin Chemical Technology Co., Ltd., Huzhou, Zhejiang, China. Nitrogen (N_2_) was provided by Hangzhou Special Gas Supply Station, Hangzhou, Zhejiang, China. Modifier A (polyester-type, Mn = 3600 g mol^−1^) was obtained from Nanjing Ningyuze Industry and Trade Co., Ltd., Nanjing, Jiangsu, China. Nano titanium dioxide (TiO_2_) was purchased from Shanghai Aladdin Biochemical Technology Co., Ltd., Shanghai, China. Sodium silicate was sourced from Shanghai Eon Chemical Technology Co., Ltd., Shanghai, China. Hydrochloric acid (HCl) was obtained from Huadong Medicine Co., Ltd., Hangzhou, Zhejiang, China. Sodium hydroxide (NaOH) was purchased from Anhui Zesheng Technology Co., Ltd., Hefei, Anhui, China. Textile detergent was supplied by Hangzhou Transfar Fine Chemicals Co., Ltd., Hangzhou, Zhejiang, China. All reagents were used as received without further purification unless otherwise specified. Poly pongee fabric (210T) was obtained from Suzhou Luliang Textile Co., Ltd., Suzhou, Jiangsu, China. Polyester taffeta fabrics (210T and 380T) were sourced from Suzhou Gelong Textile Co., Ltd., Suzhou, Jiangsu, China. Polyamide (nylon) fabric (70D) was purchased from Guangdong Zhenyuan Textile Co., Ltd., Guangzhou, Guangdong, China. Plain-woven cotton fabric (40s yarn count) was provided by Linfeng Textile Merchant Store, Haizhu District, Guangzhou, Guangdong, China.

### 2.2. Synthesis of A-PTMG/PCDL-WPU

**Scheme 1:**
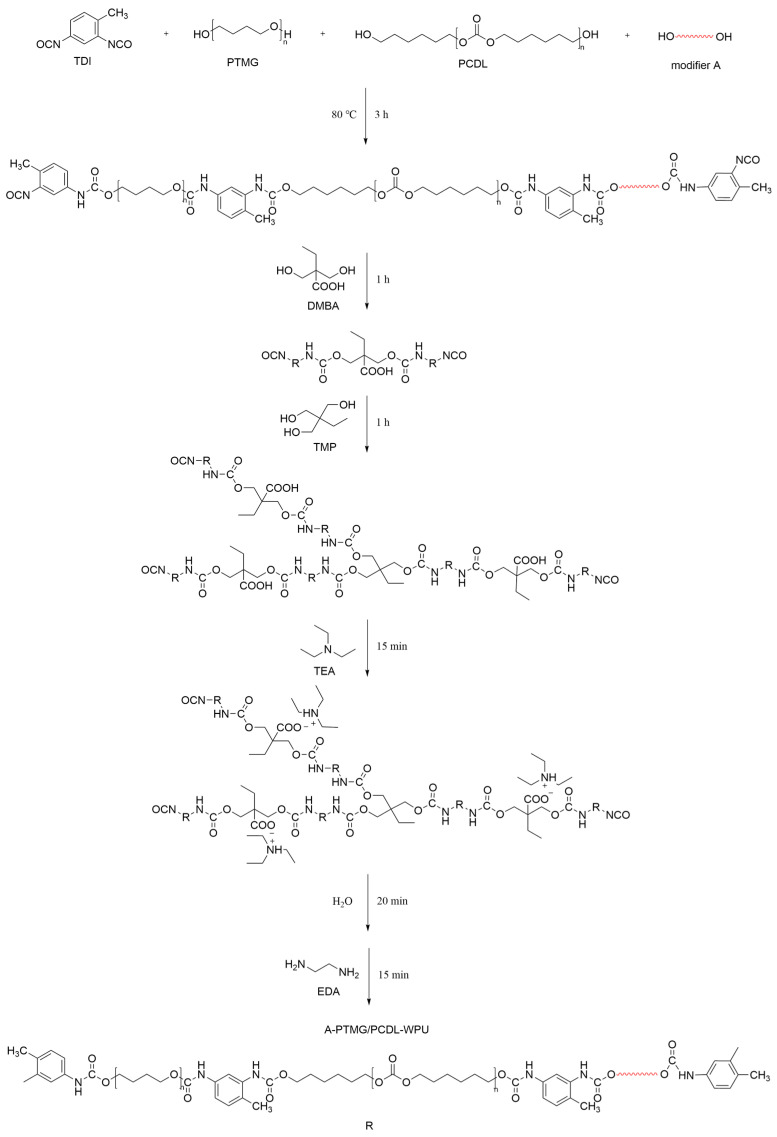
Synthesis of A-PTMG/PCDL-WPU.

PTMG and PCDL with a molar ratio of 3:7 (PTMG:PCDL) were used as the mixed soft segments. Detailed optimization procedures and performance comparisons are provided in the Supporting Information (SI). PTMG and PCDL were placed in a vacuum drying oven at 120 °C and −0.1 MPa for 6 h of vacuum dehydration. After cooling to room temperature, 33.06 g PTMG, 77.14 g PCDL, 52.52 g TDI-80, and 20.88 g modifier A were added to a dry four-neck flask. Under nitrogen protection, the mixture was heated to 80 °C and reacted for 3 h. Then, 11 g DMBA were added as a hydrophilic chain extender. After reacting at 80 °C for 1 h, 8.26 g TMP were added, and the reaction continued for another hour. The mixture was cooled to 40 °C, and 6.79 g TEA were added, reacting for 15 min. Subsequently, 300 g deionized water was rapidly added, and the mixture was emulsified under high-speed shear for 20 min. Then, 1.02 g EDA was slowly added, and the mixture was dispersed at high speed for 2 h at 40 °C. Acetone was removed under reduced pressure to obtain the modified anionic aqueous polyurethane (A-PTMG/PCDL-WPU). The synthetic route of A-PTMG/PCDL-WPU is illustrated in Scheme 1. To control the reaction viscosity, 40 g of acetone was added portionwise at each stage, concurrently with the introduction of DMBA, TMP, and TEA, respectively.

### 2.3. Synthesis of SiO_2_/TiO_2_-WPU

In total, 2.00 g TiO_2_, 0.51 g sodium hexametaphosphate, and 100 g deionized water were added to a dry four-neck flask. After high-speed stirring for 0.5 h, the mixture was heated to 90 °C. The pH was adjusted to 9–10 using 1 mol·L^−1^ NaOH solution. To deposit amorphous SiO_2_ onto the TiO_2_ surface, 0.35 g of sodium silicate was then slowly added as the silica precursor. Under the alkaline condition, the silicate hydrolyzes and condenses onto the TiO_2_ surface, forming an amorphous SiO_2_ coating. The pH was maintained at ≈10 with 0.1 mol·L^−1^ HCl, and the reaction continued at 90 °C for 1 h, followed by aging for 24 h. The product was centrifuged, washed several times with deionized water, dried at 80 °C to a constant weight, and ground into a fine powder to obtain SiO_2_-modified TiO_2_ nanoparticles. The as-prepared SiO_2_/TiO_2_ powder was added at a loading of 0.2 wt% (relative to the total mass of the A-PTMG/PCDL-WPU emulsion) and was added into the A-PTMG/PCDL-WPU emulsion, followed by ultrasonic dispersion for 60 min at room temperature to obtain the homogeneous WPU composite coating.

### 2.4. Preparation of Waterborne Polyurethane Film

The mixture was poured into a groove on a clean PTFE plate, scraped with a glass rod to form a uniform thin layer, left at room temperature for 24 h, then dried at 120 °C for 20 min to produce a bubble-free, smooth polyurethane film (0.2–0.4 mm thick).

### 2.5. Preparation of WPU-Coated Fabrics

Fabrics (Poly pongee fabric (210T), Polyester taffeta fabric (210T and 380T), Nylon fabric (70D), Cotton fabric (40s yarn count)) were immersed in a waterproofing agent for 1 min, rolled to a retention rate of 60 ± 5%, and then fixed on a needle board frame. They were dried at 130 °C for 5 min. WPU was then applied evenly with a squeegee (dry gain ≈ 60 g·m^−2^), and the fabric was heat-treated at 170 °C for 5 min, then cooled to room temperature.

### 2.6. Characterization

The infrared spectrum was measured using a Nicolet IS10 Fourier Transform Infrared Spectrometer (FT-IR) from Thermo Fisher Scientific, Waltham, MA, USA. The solid sample was ground into a fine and uniform powder using a mortar, and then tested in ATR mode. The wave-number acquisition range spanned from 400 to 4000 cm^−1^, with 32 scans and a resolution of 4 cm^−1^.

The X-ray diffraction pattern was obtained using an X-ray diffractometer (XRD) of model D8Quest from Bruker AXS GmbH, Karlsruhe, Germany. The diffraction pattern was obtained by irradiating with Cu-Kα (wavelength λ = 0.15406 nm) radiation, filtered through a graphite filter, under the conditions of an instrument operating at a voltage of 40 KV and a current of 30 mA. The thin-film or powder sample is placed on a quartz XRD stage and scanned from 5° to 50° at a scanning speed of 2°∙min^−1^.

The particle size was measured using a nanoparticle size analyzer of model SZ-100V2 from HORIBA, Kyoto, Japan. 2–3 drops of the emulsion were taken into a cuvette and diluted with deionized water to the scale value, and then the particle size distribution was tested.

Thermogravimetric analysis (TGA) was measured using a NETZSCH TG 209F1 thermogravimetric analyzer from NETZSCH, Selb, Germany. Film samples prepared from the emulsion were dried in a vacuum drying oven for 8 h. Samples weighing 1–5 mg were heated from 20 to 600 °C at a heating rate of 20 °C min^−1^ under a nitrogen flow of 50 mL min^−1^.

The UV–Vis absorption spectra of the modified WPU emulsion were measured using a UV-vis spectrophotometer with the model number UV-2600 from Shimadzu Corporation, Kyoto, Japan.

The contact angle of the modified WPU films was measured using a water droplet angle tester with the model number HYK-J08A from Dongguan Hongjin Testing Instrument Co., Ltd., Dongguan, China.

The microstructure of the fabric’s surface morphological features was observed using a scanning electron microscope (SEM) with the model number HITACHI S 4700 from Hitachi, Ltd., Tokyo, Japan.

The water absorption was measured using 2 × 2 cm samples with thicknesses ranging from 0.2 to 0.4 mm. The samples were cut and dried in a 60 °C oven until a constant mass (W_0_) was achieved. Thereafter, they were immersed in distilled water at room temperature for 24 h, dried, and weighed again (W_1_). The water absorption was calculated using the formula: water absorption = (W_1_ − W_0_)/W_0_.

The tensile strength and elongation at break of the modified WPU films were determined using an RGT-20A mechanical testing machine from Shenzhen Reger Instrument Equipment Co., Ltd., Shenzhen, China. All specimens (150 × 20 × 0.5 mm) were tested in quintuplicate, and the average values were reported according to GB/T 1040.3-2006.

The hydrostatic pressure resistance of the WPU-coated fabrics was measured in accordance with the ISO 811:2018 [31], using a hydrostatic pressure tester of model OM-1200 from Dongguan Oumei Aolan Testing Equipment Co., Ltd., Dongguan, China. The test specimens were the WPU-coated fabrics prepared strictly following the procedure described in Section 2.5. A steadily increasing water pressure was applied to one face of the fabric specimen, and the hydrostatic pressure resistance value was recorded as the pressure at which water penetration occurred at three distinct points of the test sample.

Data analysis and graph plotting were performed using Origin 2021 (OriginLab Corporation, Northampton, MA, USA). All quantitative performance tests accompanied by error bars were conducted in quintuplicate, and the corresponding mean values were reported. The error bars represent the standard error of the mean (SEM) calculated from five parallel determinations.

## 3. Results

### 3.1. FT−IR Analysis of A-PTMG/PCDL-WPU

**Figure 1:**
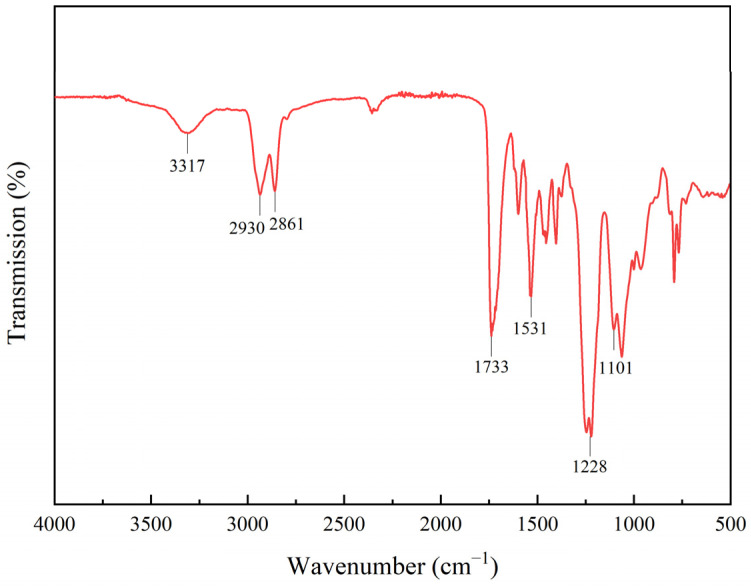
FT−IR spectrum of A-PTMG/PCDL-WPU.

The FTIR spectrum of the modified anionic waterborne polyurethane is shown in Figure 1. Only a very weak residual absorption peak can be observed at approximately 2240 cm^−1^, suggesting that the -NCO groups were almost entirely consumed in the anionic waterborne polyurethane system. The peak at 3317 cm^−1^ corresponds to the stretching vibration of the secondary amine (N-H) bond within the urethane groups, which are characteristic of polyurethane. The peaks observed at 2930 cm^−1^ and 2861 cm^−1^ are attributed to the stretching vibrations of methylene (-CH_2_) and methyl (-CH_3_) groups. The absorption peak at 1733 cm^−1^ is assigned to the carbonyl (C=O) groups. The peak at 1531 cm^−1^ arises from the bending vibration of the N-H bond in the urethane groups. The peak at 1228 cm^−1^ is associated with the C-O absorption of the ester groups, while the peak at 1101 cm^−1^ corresponds to the absorption of aliphatic ether bonds (C-O-C). The results collectively confirm the successful synthesis of the modified anionic waterborne polyurethane.

### 3.2. Crystal Structure of SiO_2_/TiO_2_ Nanoparticles

**Figure 2:**
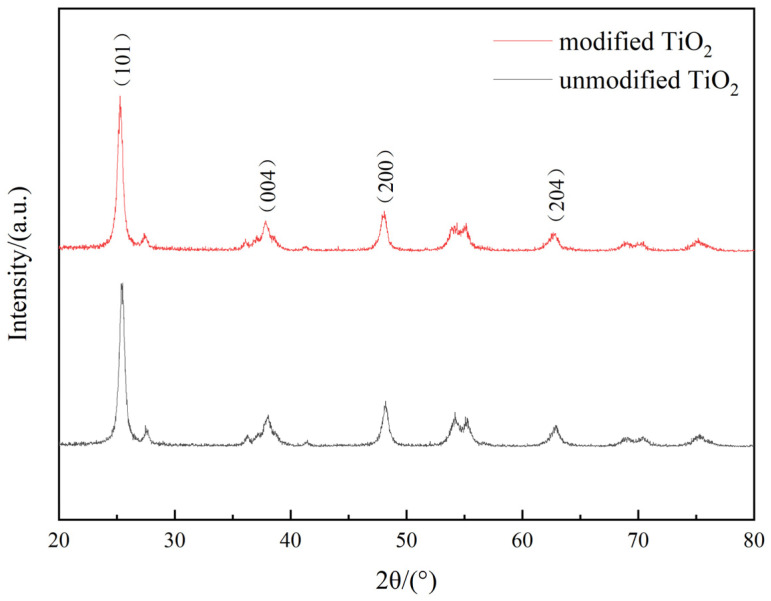
XRD spectra of TiO_2_ before and after modification with SiO_2_.

As shown in Figure 2, both pristine and SiO_2_-modified TiO_2_ exhibit characteristic diffraction peaks at approximately 25.3°, 37.8°, 48.0°, and 62.7°, corresponding to the (101), (004), (200), and (204) crystal planes of anatase TiO_2_ (JCPDS: 21-1272), respectively. No obvious shift in these TiO_2_ diffraction peaks is observed, indicating that silicon species are not doped into the TiO_2_ lattice but exist as a surface coating. Meanwhile, no characteristic diffraction peaks of crystalline SiO_2_ and no broad diffuse peak at 2θ = 20–25° for amorphous SiO_2_ is detected. This absence is attributed to the ultra-thin thickness and low content of the amorphous SiO_2_ coating layer on TiO_2_ nanoparticles, whose weak diffraction signal is fully covered by the strong diffraction peaks of anatase TiO_2_. These results confirm that the crystal form of TiO_2_ remains anatase after modification. No characteristic diffraction peaks of crystalline SiO_2_ are detected, indicating that SiO_2_ is present in an amorphous form rather than as bulk crystalline phase. Based on the sol–gel synthesis method conducted in alkaline condition with sodium silicate, it is reasonable to infer that amorphous SiO_2_ is deposited onto the TiO_2_ surface, resulting in SiO_2_-modified TiO_2_ particles.

### 3.3. Sedimentation Stability of A-PTMG/PCDL-WPU Modified by SiO_2_/TiO_2_

**Figure 3:**
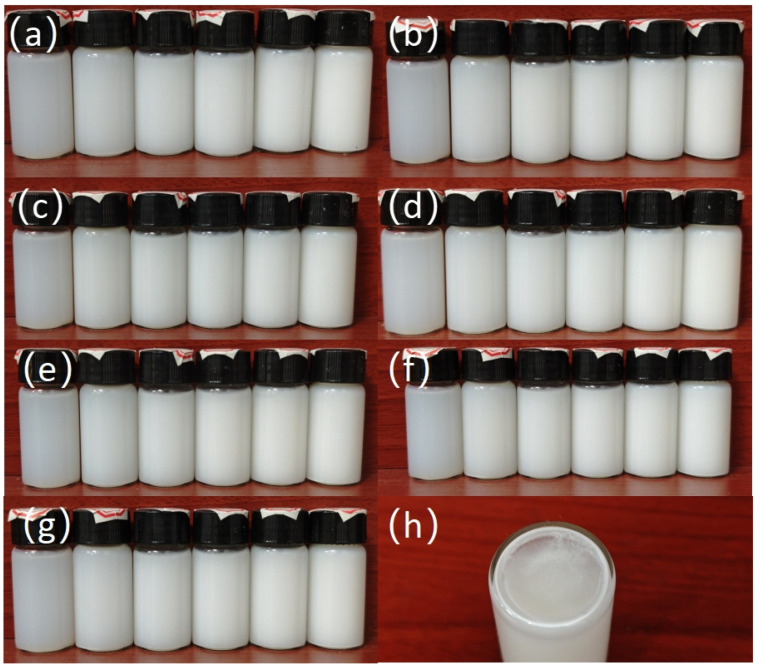
Effect of SiO_2_/TiO_2_ content on sedimentation stability of WPU. From left to right: 0, 0.1, 0.2, 0.3, 0.4, and 0.5 wt% SiO_2_/TiO_2_. (**a**–**g**) Appearance after standing for 24, 48, 72, 96, 120, 144, and 168 h, respectively. (**h**) Bottom sediment of the 0.5 wt% sample after 168 h.

As depicted in Figure 3, the appearance of the WPU emulsion changed from milky white with a bluish tint to milky white after SiO_2_/TiO_2_ incorporation, indicating increased particle size. The particle size of the WPU emulsions with different SiO_2_/TiO_2_ loadings is summarized in Table 1. With the increase in SiO_2_/TiO_2_ dosage, the particle size of the composite emulsion increased gradually from 230 nm to over 400 nm at 0.5 wt% loading. At 0.1–0.4 wt% SiO_2_/TiO_2_, the emulsion maintained a relatively uniform particle size and remained stable for 168 h. In contrast, obvious precipitation occurred at 0.5 wt% loading, which was mainly attributed to the excessively large particle size, aggravated interparticle steric hindrance and van der Waals forces.

### 3.4. UV-Vis Absorption Spectra of Modified WPU Emulsions

**Figure 4:**
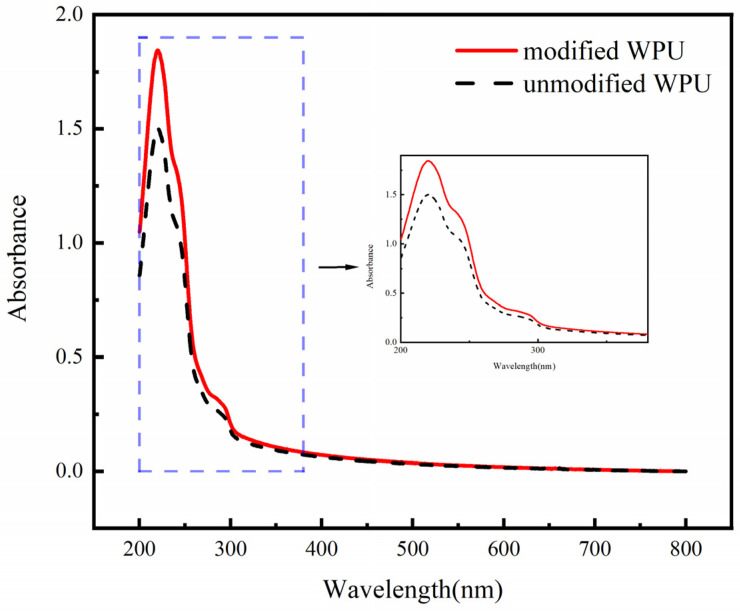
UV absorption spectra of unmodified and SiO_2_/TiO_2_-modified WPU emulsions. The blue square indicates the magnified region shown in the inset.

The modified and unmodified WPU were prepared into a dispersion solution with a concentration of 50 mg·L^−1^, and we conducted UV absorption spectroscopy tests on them. As shown in Figure 4, both modified and unmodified WPU exhibit effective absorption in the ultraviolet region of 200–380 nm, and the absorption intensity of the modified WPU is significantly higher than that of the unmodified one. The enhanced UV absorption intensity is attributed to the intrinsic ultraviolet absorption characteristic of anatase TiO_2_ in SiO_2_/TiO_2_, which originates from the electron transition from the O 2p valence band to the Ti 3d conduction band in TiO_2_. Meanwhile, the absorption peak shape of the modified WPU remains almost consistent with that of the unmodified sample. This is because SiO_2_/TiO_2_ is physically blended into the WPU matrix without changing the molecular structure of WPU, and TiO_2_ presents a broad and continuous absorption band in the ultraviolet region without new characteristic peaks. As a result, only the absorption intensity is enhanced while the peak shape remains unchanged, which not only confirms that SiO_2_/TiO_2_ is successfully introduced into the composite system as a filler, but also indicates an enhanced UV absorption capacity that may be relevant for UV-protective applications.

### 3.5. Physicochemical and Functional Properties of Modified WPU Films

#### 3.5.1. Thermal Decomposition Temperatures of Modified WPU Films

**Figure 5:**
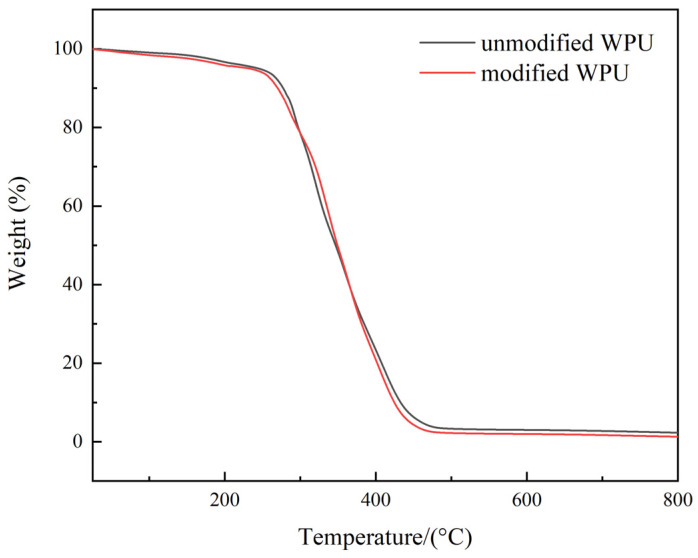
Thermal decomposition temperatures of WPU films before and after modification with SiO_2_/TiO_2_.

The prepared A-PTMG/PCDL-WPU and SiO_2_/TiO_2_-A-PTMG/PCDL-WPU films were subjected to thermogravimetric analysis (TGA), and the results are presented in Table 2 and Figure 5. After the addition of SiO_2_/TiO_2_, the 10% mass loss temperature (T_10_%) and 90% mass loss temperature (T_90_%) of the films decreased from 274.36 °C and 430.14 °C to 270.03 °C and 424.57 °C, respectively, while the 50% mass loss temperature (T_50_%) slightly increased. The decrease in T_10_% is attributed to the fact that some TiO_2_ surfaces were not fully covered by SiO_2_, leaving catalytically active TiO_2_ surfaces exposed at low temperatures and promoting the degradation of the WPU segment. The decrease in T_90_% is likely due to the degradation of the SiO_2_ surface coating at high temperatures, re-exposing the underlying TiO_2_ to catalyze the decomposition of the matrix. The temperature increase at T_50_% is attributed to the combined hindrance of heat transfer by the uniformly dispersed SiO_2_/TiO_2_ particles and the residual carbon layer generated by pyrolysis.

#### 3.5.2. Mechanical Performance of Modified WPU Films

**Figure 6:**
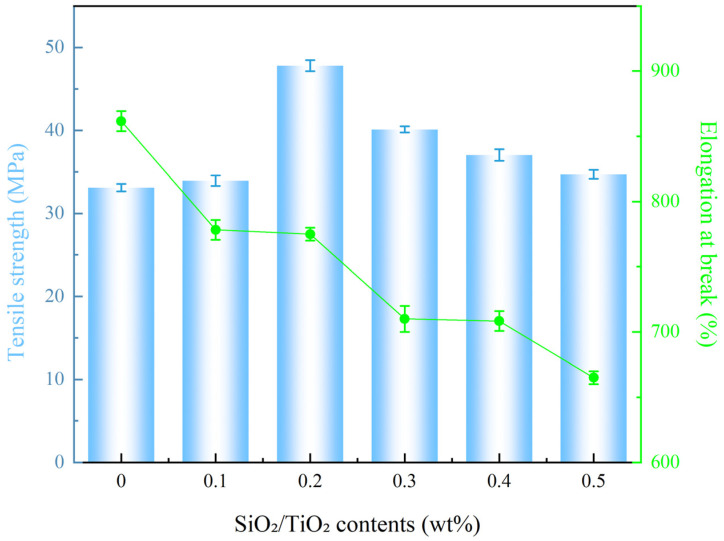
The impact of SiO_2_/TiO_2_ dosage on the tensile strength and elongation at break of WPU films.

The tensile properties of WPU films with varying SiO_2_/TiO_2_ contents are presented in Figure 6. As the SiO_2_/TiO_2_ content is increased, the elongation at break is observed to decrease continuously, while the tensile strength is found to initially increase and subsequently decrease. When the SiO_2_/TiO_2_ content is 0.2 wt%, the tensile strength reaches 47.29 MPa, and the elongation at break is 780%. The tensile strength of the WPU film without SiO_2_/TiO_2_ is 32.66 MPa, and the elongation at break is 860%. This is because SiO_2_/TiO_2_, as a rigid material, restricts the movement of WPU segments upon its introduction, and hydrogen bonds can form between the SiO_2_ surface and WPU molecular segments, thereby enhancing the tensile strength of the WPU film. Correspondingly, the restricted movement of WPU segments weakens the ductility of the WPU film, ultimately manifesting as a decrease in elongation at break.

#### 3.5.3. Water Resistance of Modified WPU Films

**Figure 7:**
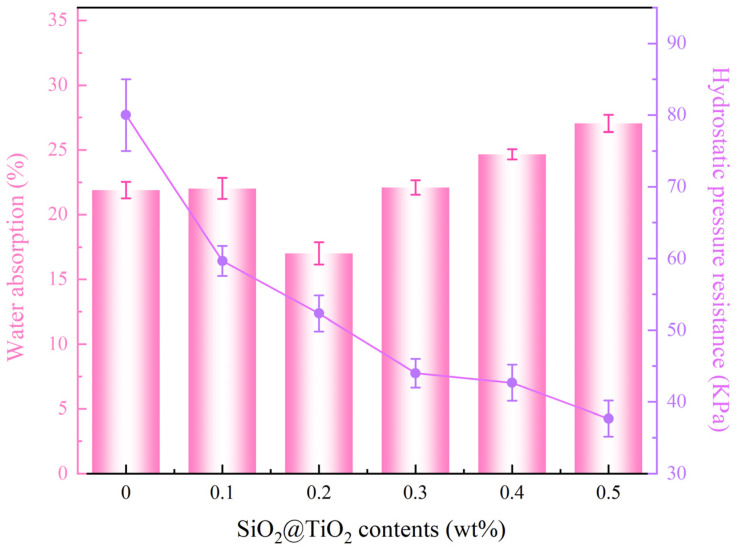
The impact of SiO_2_/TiO_2_ dosage on the water absorption rate and hydrostatic pressure resistance of WPU films.

As depicted in Figure 7, the water absorption rate of the WPU film initially decreases and subsequently increases with increasing SiO_2_/TiO_2_ content, whereas the hydrostatic pressure resistance exhibits a monotonic decline. In the absence of SiO_2_/TiO_2_, the water absorption rate is 21.48% and the hydrostatic pressure resistance reaches 80 kPa. At a SiO_2_/TiO_2_ loading of 0.2 wt%, the water absorption rate reaches its minimum value of 16.11%, and the hydrostatic pressure resistance remains at a relatively high level. Further increasing the content to 0.5 wt% causes the water absorption rate to rebound to 26.55%, accompanied by a continuous drop in hydrostatic pressure resistance to 38 kPa. Such a trend can be explained by the dual effects of SiO_2_/TiO_2_. On the one hand, uniformly dispersed SiO_2_/TiO_2_ at low loadings effectively fills the internal micropores of the WPU film, which helps reduce the water absorption rate. On the other hand, the continuous reduction in hydrostatic pressure resistance arises from the inherent hydrophilicity of SiO_2_/TiO_2_ and the induced structural defects. Even at low dosages, the hydroxyl-rich SiO_2_ surface introduces hydrophilic sites into the WPU film, and the rigid particles tend to form interfacial micro-voids that act as preferential pathways for water penetration under pressure. The large number of hydroxyl groups on the SiO_2_ surface enhances water adsorption via hydrogen bonding, while the aggravated defects further accelerate water permeation. These factors collectively lead to the rebound in water absorption and the steady decline in hydrostatic pressure resistance. It should be noted that the decreased hydrostatic pressure resistance of the freestanding film does not limit its practical application. When coated onto tightly woven fabrics, the supporting substrate can effectively compensate for the structural defects at the film level, thus maintaining satisfactory waterproof performance for textile applications.

### 3.6. Morphology and Comprehensive Performance of WPU-Coated Fabrics

#### 3.6.1. SEM Image of WPU-Coated Fabrics

**Figure 8:**
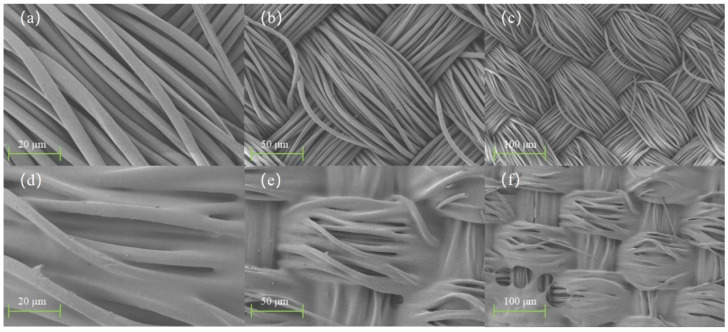
SEM micrographs of uncoated and coated Poly pongee fabrics (210T) at different magnifications. (**a**–**c**) Blank fabric; (**d**–**f**) Coated fabric. Scale bars: 20 μm, 50 μm, and 100 μm, respectively.

Scanning electron microscopy was conducted on both the blank Poly pongee fabrics and the coated Poly pongee fabrics to examine their surface morphological features. As evident from Figure 8, in contrast to the blank fabric, the coated fabric exhibited the formation of a continuous WPU film on its surface, characterized by a smooth texture, fiber adhesion, and diminished friction.

#### 3.6.2. The Effect of Incorporating SiO_2_/TiO_2_ on the Static Water Contact Angle of WPU-Coated Fabrics

**Figure 9:**
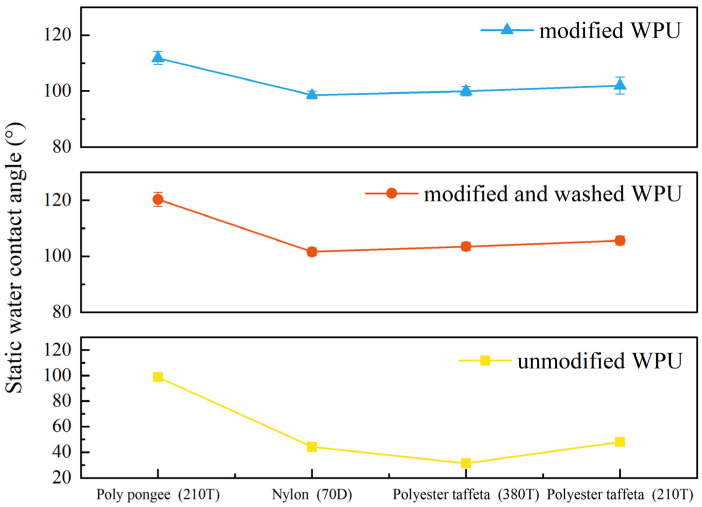
Static water contact angles of different fabrics under three treatment conditions: untreated, coated with SiO_2_/TiO_2_-modified WPU, and coated followed by standard washing.

Static water contact angle measurements were performed on various fabrics coated with unmodified WPU, modified WPU, as well as on those coated with modified WPU and then subjected to laundering. The related results in Figure 9 demonstrate that the static water contact angles of fabrics coated with modified WPU exhibit a substantial increase compared to their uncoated counterparts. This enhancement is attributed to the hydrophobic characteristics conferred upon the fabric surface by the modified WPU coating, leading to an enlarged water contact angle. Following laundering, the static water contact angles of fabrics coated with modified WPU demonstrate a decrease relative to those that have not undergone laundering, owing to the partial degradation of the WPU coating caused by the washing process. Nevertheless, with the exception of cotton fabrics, all other fabric types retained static water contact angles exceeding 90°.

#### 3.6.3. The Effect of Incorporating SiO_2_/TiO_2_ on the Hydrostatic Pressure Resistance of WPU-Coated Fabrics

**Figure 10:**
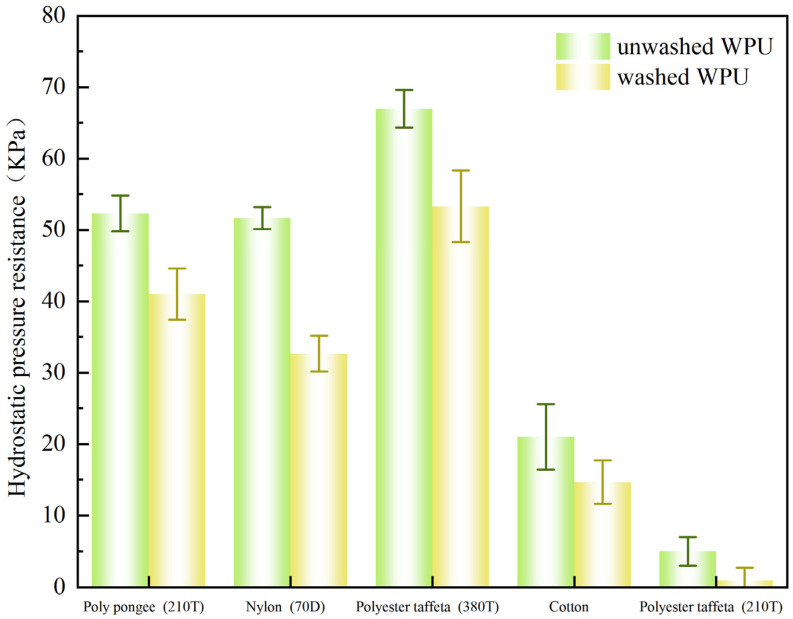
Hydrostatic pressure resistance of modified WPU on different fabrics.

After applying modified WPU coating to four distinct types of fabrics, the hydrostatic pressure resistance was evaluated. It is revealed in Figure 10 that following treatment with modified WPU, the extent of improvement in hydrostatic pressure resistance exhibits a positive correlation with the fabric’s structural density: high-woven-density fabrics, exemplified by 380T Polyester taffeta fabrics demonstrate a pronounced barrier effect, leading to a marked enhancement in hydrostatic pressure resistance; conversely, 210T Polyester taffeta fabrics characterized by its sparse structure and elevated porosity, fail to establish a continuous and compact barrier even after being coated with modified WPU, thereby resulting in significantly lower hydrostatic pressure resistance. Subsequent to standard water washing, the coating integrity diminishes, and the waterproofing capability of each fabric undergoes varying degrees of degradation, with the most loosely woven 210T Polyester taffeta fabrics experiencing near-total loss of effectiveness. In contrast, for 380T polyester taffeta, the hydrostatic pressure resistance decreased from 66.00 kPa to 54.00 kPa after standard washing, retaining satisfactory waterproof performance.

### 3.7. Comparison with Reported Waterborne Polyurethane Materials

Collectively, the SiO_2_/TiO_2_-A-PTMG/PCDL-WPU composite film at the optimal 0.2 wt% loading of SiO_2_/TiO_2_ exhibits superior mechanical performance (tensile strength: 47.29 MPa) and low water absorption (16.11%). However, the hydrostatic pressure resistance of freestanding films decreases monotonically with increasing filler loading. Nevertheless, when coated onto high-density 380T polyester taffeta, the fabric retains a satisfactory hydrostatic pressure resistance of 66.00 kPa. To further systematically evaluate the comprehensive performance of the material developed in this work, Table 3 compares its key metrics (hydrostatic pressure resistance, tensile strength, elongation at break, water absorption) with those of other WPU-based materials reported in the recent literature.

## 4. Conclusions

In this paper, SiO_2_/TiO_2_ nanoparticles were physically blended with modifier A-modified PTMG/PCDL-based waterborne polyurethane (WPU). The optimized molar ratio of PTMG to PCDL (3:7) acts as a crucial structural prerequisite for the composite films to attain outstanding overall performance, as such a balanced composition provides robust structural support and endows the films with excellent mechanical properties, water resistance, and hydrostatic pressure resistance. Based on the experimental results, the following conclusions can be drawn: The introduction of modifier A into the WPU prepolymerization system significantly enhanced the hydrostatic pressure resistance of the films while maintaining a high elongation at break. The composite WPU film achieved the optimal comprehensive performance at a SiO_2_/TiO_2_ loading of 0.2 wt%, exhibiting a tensile strength of 47.29 MPa, an elongation at break of 780%, and a water absorption rate of 16.11%. Moreover, the SiO_2_/TiO_2_/WPU composite coating showed enhanced UV absorption and improved surface water repellency to various fabrics, while the composite film itself displayed enhanced mechanical strength. While the hydrostatic pressure resistance of the film showed a decreasing trend with increasing filler content, the composite coating on high-density 380T polyester taffeta still achieved satisfactory hydrostatic pressure resistance (66.00 kPa) and retained 54.00 kPa after standard washing, confirming its excellent washfastness and promising practical applicability.

## Figures and Tables

**Table 1:** Particle size of WPU emulsions with different SiO_2_/TiO_2_ loadings.

SiO_2_/TiO_2_ Content (wt%)	Particle Size (nm)
0	230
0.1	240
0.2	255
0.3	275
0.4	300
0.5	>400 (precipitation)

**Table 2:** Characteristic thermal decomposition temperatures of A-PTMG/PCDL-WPU and SiO_2_/TiO_2_-modified WPU films.

Sample Name	T_10%_/°C	T_50%_/°C	T_90%_/°C
A-PTMG/PCDL-WPU	274.36	344.04	430.14
SiO_2_/TiO_2_-A-PTMG/PCDL-WPU	270.03	349.21	424.57

**Table 3:** Comparison of comprehensive properties between this work and representative WPU-based materials reported in the literature.

Ref.	Key Modification/Strategy	Substrate/Type	Hydrostatic Pressure (kPa)	Tensile Strength (MPa)	Elongation at Break (%)	Water Absorption (%)
This work	SiO_2_/TiO_2_ physical blending	380T polyester taffeta	66.00 (coated fabric)	47.29	780	16.11
[22]	Itaconic acid ester grafting	WPU-coated fabric	18.9	8.61	325	17.4
[24]	Long-chain alkyl polymer	WPU nanofibrous membrane	35.9	2.3	372.4	NR
[25]	GO-CNTs physical blending	WPU film	147.5	5.6	280	NR

Note: NR = not reported.

## Data Availability

The original contributions presented in the study are included in the article and Supplementary Material. Further inquiries can be directed to the corresponding author.

## Supplementary Materials

The following supporting information can be downloaded at: https://www.mdpi.com/article/10.3390/polym18121492/s1, Table S1: Effects of PTMG/PCDL molar ratio on the properties of WPU films; Table S2: Effects of modifier A dosage on the properties of WPU emulsions and films.
