# Supplementary material for: Waterborne Polyurethane Reinforced with SiO2-Modified TiO2: Enhanced Mechanical Properties and Retained Hydrostatic Pressure Resistance
Source: Polymers (Basel). 2026 Jun 13;18(12):1492. doi: 10.3390/polym18121492 (PMC13307451; doi:10.3390/polym18121492)
Supplement: Supplementary file 1 [file polymers-18-01492-s001.zip › polymers-4336119-supplementary.pdf]

Supplementary Materials for

SiO_2@TiO_2-Modified Waterborne Polyurethane with Enhanced Mechanical Properties and Retained Hydrostatic Pressure Resistance

S1 Optimization of PTMG/PCDL Soft Segment Ratio

S1.1 Experimental Method

A series of waterborne polyurethane (WPU) emulsions with different PTMG/PCDL molar ratios (0:10, 1:9, 3:7, 5:5, 7:3, 9:1, 10:0) were synthesized to determine the optimal soft segment ratio. The mechanical properties, water absorption rate and hydrostatic pressure resistance of the WPU films were tested to evaluate the comprehensive performance.

S1.2 Results and Performance Comparison

Table S1. Effects of PTMG/PCDL molar ratio on the properties of WPU films

PCDL:PTMG	Appearance	Particle Size (nm)	Tensile Strength (MPa)	Elongation at Break (%)
0:10	White, bluish	62.2	38.12	940
1:9	White, bluish	62.3	43.91	880
3:7	White, bluish	66.1	46.37	860
5:5	White, bluish	66.4	47.19	860
7:3	Yellowish, translucent	66.5	52.89	640
9:1	Yellowish, translucent	66.9	53.58	540
10:0	Yellowish, translucent	93.3	55.96	460

S1.3 Advantages of the Optimal Ratio (PCDL:PTMG = 7:3)

1.Mechanical balance: PTMG provides high elongation and flexibility, while PCDL enhances tensile strength. The 7:3 ratio avoids excessive brittleness (high PCDL) and insufficient strength (high PTMG).

2.Water resistance improvement: The polar carbonate groups in PCDL form stronger intermolecular hydrogen bonds, leading to more compact chain stacking,and reduce water absorption; the ether segments in PTMG maintain film integrity and prevent cracking.

3.Comprehensive optimal performance: The 3:7 ratio achieves the best balance of mechanical properties, water resistance and hydrostatic pressure resistance, which is suitable for subsequent modification and fabric coating.

S2 Optimization of Modifier A Dosage for High Hydrostatic Pressure Resistance

S2.1 Experimental Method

Based on the optimal base WPU formulation (prepolymer R-value=2.6, PCDL:PTMG=7:3, DMBA=6 wt%, TMP=4.5 wt%, TEA=90%, EDA=90%), a series of modifier A-modified WPU emulsions were synthesized by introducing polyester-based modifier A (Mn=3600) at different dosages (1 mol%, 3 mol%, 5 mol%, 7 mol%, 9 mol%, calculated as the molar ratio of hydroxyl groups in modifier A to total hydroxyl groups in the prepolymer system) during the prepolymerization stage. The emulsion stability, mechanical properties and hydrostatic pressure resistance of the modified WPU films were tested to determine the optimal modifier A dosage.

S2.2 Results and Performance Comparison

Table S2. Effects of modifier A dosage on the properties of WPU emulsions and films

Modifier A dosage (mol%)	Appearance	Particle Size (nm)	Tensile Strength (MPa)	Elongation at Break (%)	Hydrostatic pressure resistance (kPa)
0	Yellowish, translucent	66.5	52.89	640	14
1	White, bluish	101.8	50.65	660	26

3	White, bluish	114.9	40.51	740	58
5	Semitransparent, bluish white	85.5	32.66	860	80
7	Semitransparent, bluish white	59.6	29.51	940	45
9	Semitransparent, bluish white	45.7	20.29	1020	22

S2.3 Performance Advantages of the Optimal Modifier A Dosage (5 mol%)

1.Significant enhancement of hydrostatic pressure resistance: The hydrostatic pressure resistance increased from 14.00 kPa to 80.00 kPa, a 5.7-fold improvement. This is attributed to the introduction of long-chain polyester segments in modifier A, which optimized the microphase separation of soft and hard segments and formed a more continuous and defect-free soft segment domain, effectively blocking water penetration channels.

2.Maintenance and improvement of elongation at break: The elongation at break increased from 640% to 860%, maintaining excellent flexibility. The flexible polyester segments in modifier A increased the proportion of soft segments in the WPU molecular chain, enhancing the chain mobility and elasticity of the film.

3.Balanced comprehensive performance: Although the tensile strength decreased, the 5 mol% dosage achieved the best balance between hydrostatic pressure resistance and mechanical flexibility, which is suitable for fabric coating applications requiring both waterproof performance and wear comfort.
